# Potential of a no‐take marine reserve to protect home ranges of anadromous brown trout (*Salmo trutta*)

**DOI:** 10.1002/ece3.4760

**Published:** 2018-12-18

**Authors:** Susanna Huneide Thorbjørnsen, Even Moland, Colin Simpfendorfer, Michelle Heupel, Halvor Knutsen, Esben Moland Olsen

**Affiliations:** ^1^ Center for Coastal Research, Department of Natural Sciences University of Agder Kristiansand Norway; ^2^ Institute of Marine Research Flødevigen Norway; ^3^ Centre for Sustainable Tropical Fisheries and Aquaculture & College of Science and Engineering James Cook University Townsville Queensland Australia; ^4^ Australian Institute of Marine Science Townsville Queensland Australia

**Keywords:** acoustic telemetry, behavior, marine reserve design, movement, sea trout, selection, space use

## Abstract

The extent to which no‐take marine reserves can benefit anadromous species requires examination. Here, we used acoustic telemetry to investigate the spatial behavior of anadromous brown trout (sea trout, *Salmo trutta*) in relation to a small marine reserve (~1.5 km^2^) located inside a fjord on the Norwegian Skagerrak coast. On average, sea trout spent 42.3 % (±5.0% *SE*) of their time in the fjord within the reserve, a proportion similar to the area of the reserve relative to that of the fjord. On average, sea trout tagged inside the reserve received the most protection, although the level of protection decreased marginally with increasing home range size. Furthermore, individuals tagged outside the reserve received more protection with increasing home range size, potentially opposing selection toward smaller home range sizes inflicted on fish residing within reserves, or through selective fishing methods like angling. Monthly sea trout home ranges in the marine environment were on average smaller than the reserve, with a mean of 0.430 (±0.0265 *SE*) km^2^. Hence, the reserve is large enough to protect the full home range of some individuals residing in the reserve. *Synthesis and applications*: In general, the reserve protects sea trout to a varying degree depending on their individual behavior. These findings highlight evolutionary implications of spatial protection and can guide managers in the design of marine reserves and networks that preserve variation in target species' home range size and movement behavior.

## 1 INTRODUCTION

Marine protected areas (MPAs) are widely used as a means to protect species in their habitat and have been shown to increase numbers and/or biomass of protected species, both inside MPAs (Lester et al., 2009) and as spillover beyond MPA borders (Abesamis & Russ, 2005; Goñi, Hilborn, Díaz, Mallol, & Adlerstein, 2010; Roberts, Bohnsack, Gell, Hawkins, & Goodridge, 2001). Efficacy of MPAs is expected to be higher for less mobile species (Pilyugin, Medlock, & Leenheer, 2016), but positive effects have also been found for wide ranging species, such as coastal sharks. For example, Knip, Heupel, and Simpfendorfer (2012) found that coastal shark species resided in an MPA 22%–32% of their time, and that the MPA provided similar protection to all size classes. MPAs can protect mobile species if strategically situated, as shown for white stumpnose (Kerwath et al., 2008) and migratory sea turtles (Hays, Mortimer, Ierodiaconou, & Esteban, 2014). Since migratory species move in predictable patterns, there is potential to recognize and protect key areas of their habitat using MPAs or strictly no‐take zones (marine reserves).

**Figure 1:**
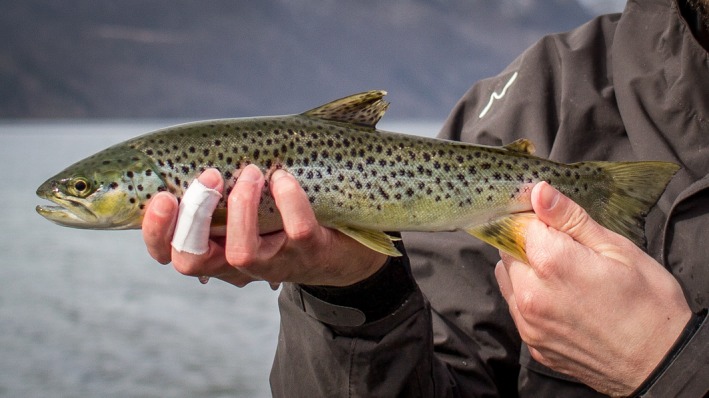
Brown trout (*Salmo trutta*). Photo: Erlend A. Lorentzen

A number of fish species are known to undertake migrations for a variety of purposes such as spawning and feeding (Block et al., 2001; Hunter, Metcalfe, & Reynolds, 2003; Klemetsen, 2003). Salmonids are often anadromous, migrating between spawning areas in fresh water (rivers) and the marine environment. Brown trout (*Salmo trutta*, Figure 1) is a salmonid species with an anadromous component called sea trout. It has a highly variable life history, with some trout spending their whole life in the river, and others spending most of their time in the marine environment (Klemetsen et al., 2003). Predicting the efficiency of marine reserves for species with highly variable migratory patterns, such as the sea trout, is a major challenge. Variation in how sea trout use marine habitats is substantial and ranges from spending only a few weeks at sea (Eldøy et al., 2015) to spending two or more years at sea (Jonsson & Jonsson, 2002; Klemetsen et al., 2003). In addition, there is great variation in habitat use in marine regions, with some sea trout spending most of their time in fjords and some venturing out to the open seas (Bordeleau et al., 2018; del Villar‐Guerra, Aarestrup, Skov, & Koed, 2014). Seaward migration can occur as a response to reduced energetic surplus available for growth (Forseth, Næsje, Jonsson, & Hårsaker, 1999) and is also more likely for individuals with a lower body condition (Bordeleau et al., 2018). Decisions made regarding staying in fjord habitats or moving to the open sea are made shortly after entering the fjord (del Villar‐Guerra et al., 2014). Additionally, sea trout may stray to rivers other than their natal river, also to spawn (Berg & Berg, 1987; Degerman, Leonardsson, & Lundqvist, 2012; Thorstad et al., 2016 and references therein). Acquiring knowledge on habitat use of sea trout in relation to a no‐take zone can assist managers in positioning of reserves and in evaluating a potential MPA network design.

A study of wild‐origin zebrafish (*Danio rerio*) revealed that size*‐*selective harvesting alters the behavioral composition in a target population, resulting in less explorative and bold individuals (Uusi‐Heikkilä et al., 2015). Angling selects against bold behavior and large home ranges (Alós, Palmer, Rosselló, & Arlinghaus, 2016; Klefoth, Skov, Kuparinen, & Arlinghaus, 2017), and one mechanism behind this is that fish that utilize larger areas and have a higher movement rate have a higher risk of encountering hooks (Enberg et al., 2012). In Norway, fishing for sea trout is mainly by hook and line, leaving sea trout vulnerable to angling‐induced selection. Marine reserves also have the potential to select against large home range size depending on an individual's home range size relative to reserve size (Villegas‐Ríos, Moland, & Olsen, 2016). Selection on behavior and movement can indirectly select on life‐history traits like growth and fecundity (Biro & Stamps, 2008) and thus alter the productivity in a population, which in turn will affect fishing yields. The interplay between these selective effects will determine how a marine reserve succeeds in protecting a population and its different behavioral components (see Baskett & Barnett, 2015).

Acoustic telemetry can be used to acquire long‐term detailed information on movement in marine animals and using a dense network of acoustic receivers allows for calculating centers of activity (Simpfendorfer, Heupel, & Hueter, 2002) and home ranges (Villegas‐Ríos, Réale, Freitas, Moland, & Olsen, 2017). We used acoustic telemetry to quantify spatial use of sea trout in a southern Norwegian fjord in relation to a no‐take marine reserve, as well as adjacent partially protected marine habitats and areas open to all types of fishing. We expected that habitat use during the marine phase would vary substantially among individual sea trout, and that the amount of protection afforded by the no‐take marine reserve would be influenced by tagging location and home range size.

## 2 MATERIALS AND METHODS

### 2.1 Study species

The brown trout (*Salmo trutta*) is a salmonid fish that spawns in fresh water and subsequently adopts various migratory strategies, with some individuals spending their whole life in fresh water and others being anadromous and undertaking marine migrations (Jonsson, 1985; Jonsson & Jonsson, 1993). Spawning occurs during autumn, and migrations are cued by river flow (Jonsson & Jonsson, 2002). The sea trout is highly valued by recreational fishers. In Norway, sea trout can be fished using hook and line equipment all year in marine locations, and traps are allowed for 1 month in summer in the southern part of Norway.

### 2.2 Study site and data collection

**Figure 2:**
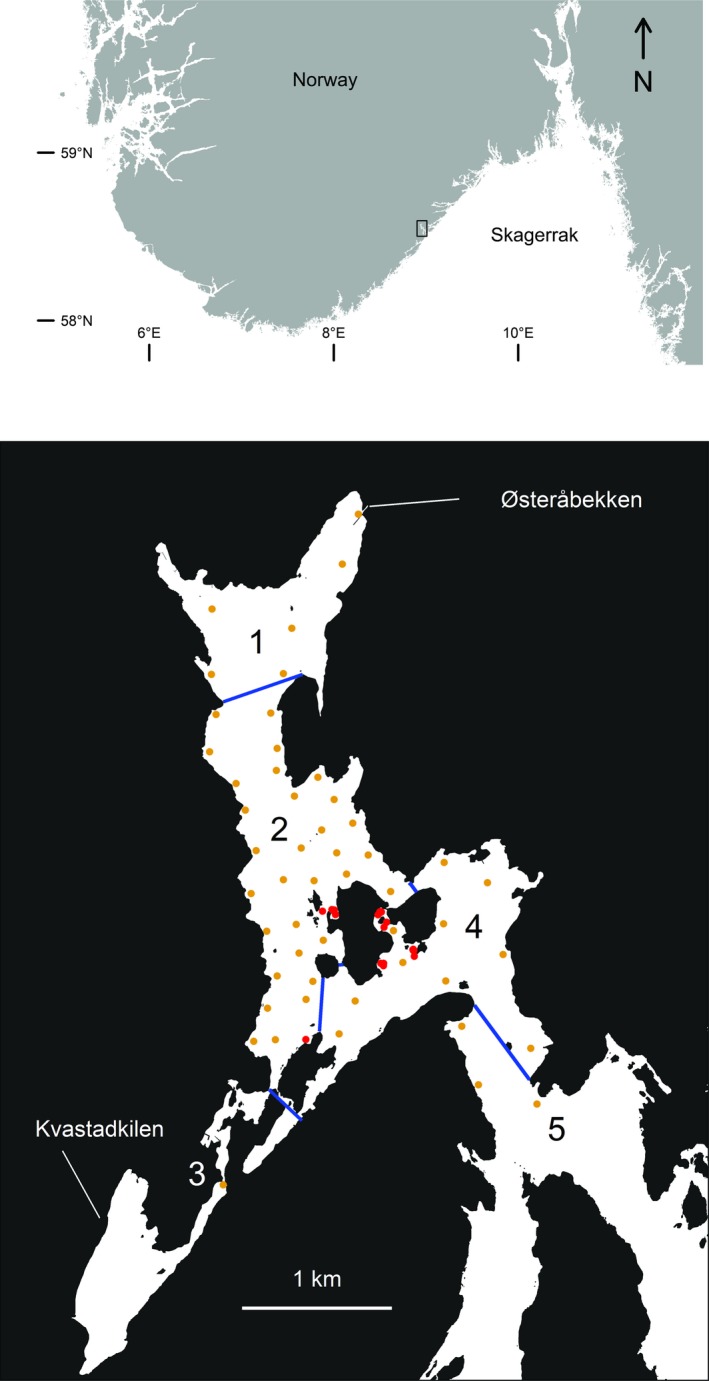
Map of the Tvedestrand fjord with zones (bottom) and its location along the Norwegian coastline (top). Red and yellow dots represent tagging and receiver locations, respectively, and blue lines section the fjord into the five different zones: The Northern MPA, including the spawning river Østeråbekken (1); the marine reserve (2); Kvastadkilen (3); the central fjord MPA (4); and the outer zone with no fishing restrictions (5)

The Tvedestrand fjord is located on the Skagerrak coast in southern Norway and covers an area of approximately 3.8 km^2^, with depths reaching 87 m. Outside the receiver array, the fjord splits into Oksefjorden and Eikelandsfjorden, which connect to the open ocean, hereby referred to as outer fjord and sea areas. A network of 50 VR2W receivers (Vemco Ltd., Halifax, Canada) was deployed in the fjord. All receivers were attached to moorings and deployed at ~3 m depth where they were kept in place by subsurface buoys. Receivers were deployed to cover most regions of the fjord, including the no‐take reserve, adjacent MPAs, and potential spawning rivers. A no‐take marine reserve designated in 2012 to protect fishes and lobsters from commercial and recreational fishing, hereafter referred to as “the reserve” (1.5 km^2^), is located in the central area of the Tvedestrand fjord (Figure 2). One receiver was deployed close to the inlet of the main spawning stream, Østeråbekken, to monitor freshwater migrations. Fish were classified as being in the river if both the last detection before an absence and the first detection after an absence occurred at the receiver in the spawning river inlet or the second closest receiver (Figure 2). One receiver was positioned to identify fish moving to the inner basin in the southwest part of the fjord (Kvastadkilen). Three receivers were located in the outermost section of the Tvedestrand fjord to identify fish movements between the fjord and the outer fjord and sea areas bordering the Skagerrak ocean. Receiver coverage was good in all zones of the fjord (see Supporting Information Figure S1).

Sea trout were caught around the center islands of the fjord using a beach seine between April and November 2013. This active fishing gear was chosen to avoid selecting individuals with a more active behavior that would potentially be favored if using angling or passive nets (Olsen, Heupel, Simpfendorfer, & Moland, 2012). Immediately following capture, individuals bigger than 23 cm were anesthetized by a 9:1 ethanol—clove oil solution added at 2 ml per 5 L of water and tagged with Vemco V9P‐L transmitters, positioned in the abdominal cavity (see Olsen et al., 2012). The transmitters were equipped with pressure sensors with an accuracy of ±2.5 m and a resolution of 0.22 m to a max depth of 50 m. Time lag between signal emissions was 120 ± 60 s and expected battery life was 550 days. The detection intervals were similar in the different zones (see Supporting Information Figure S1). All fish were released from shore at the capture location.

### 2.3 Data preparation and analyses

Detection data were downloaded from the receivers and processed using VUE software (VEMCO). An individual was defined as dead at the point where vertical and horizontal movement ceased, and the remaining data were deleted from the dataset. Detections below 50 m and single detections within 1 day were removed, as they are likely to be false. All following calculations and analyses were performed in the R environment (R Core Team, 2016). Horizontal locations were estimated using position averaging (PAV), following Simpfendorfer et al. (2002). PAVs were calculated as centers of activity for 30‐min time intervals and assigned to the appropriate fjord zone and time of day (day/night). Day and night was defined by positive and negative solar elevation, respectively. Monthly 95% home ranges (HR) for each fish were calculated from PAVs using Kernel Utilization Distributions (bandwidth = 60, extent = 0.5).

For the purpose of this study, the Tvedestrand fjord was divided into five zones: a northernmost zone comprising an MPA where no fixed gear is allowed, also including the main spawning river Østeråbekken where no fishing is allowed (Zone 1); the no‐take marine reserve (Zone 2); Kvastadkilen (Zone 3); central fjord area MPA (no fixed gear; Zone 4); and the outermost section of the Tvedestrand fjord with no restrictions (Zone 5; Figure 2). The proportion of time spent in each zone was calculated using the number of PAVs (each representing 30 min) assigned to a specific zone for both individual trout and the tagged population as a whole. In the latter case, all PAVs calculated for the tagged population were pooled.

Linear modeling was used to test if body length (mean = 0, *SD* = 1) had an effect on the proportion of time spent in the reserve. Further, to test whether home range size, tagging location (two levels: within/outside the reserve) and the interaction between these affected the proportion of time spent in the reserve, a linear mixed‐effects (lme) model was constructed based on monthly estimates of home range size, with individual as a random effect. The lme model was compared to a generalized least squares (gls) model to assess the necessity of including individual as a random effect. The model selection was based on AIC‐values, and significant improvement was assigned following a minimum reduction in two AIC units. Sizes of home ranges were log‐transformed for normality. To ensure that estimated home ranges were representative of sea trout habitat use, all months with <14 days of presence were excluded from the dataset in models including home range as a variable. A linear model fitted using generalized least squares was used to test whether season had an effect on the proportion of time spent in the reserve on a monthly basis. As sea trout spent different amounts of time within the study site in the Tvedestrand fjord, a linear model was used to test whether observation time (in months) affected the proportion of time spent in the reserve. A linear model was also used to check whether calculated home ranges were related to the number of PAVs available for a given month (Becker et al., 2016).

How often and in which direction sea trout ventured from the reserve was examined, excluding individuals that did not visit the reserve (*n* = 4). To test whether there were more excursions from the reserve during day or night, a Pearson's chi‐squared proportionality test was used. Since there were more observations during day than night, proportions were corrected accordingly by multiplying the number of detections during night by the ratio of day/night detections. The effect of body length, body condition (Fulton's *K* = 100 × Weight (g) × Length [cm]^−3^), and sex on the average daily number of excursions was also assessed by linear modeling. The effect of home range on monthly number of excursions was assessed by a lme model including individual as a random effect, and compared to a gls model to assess the necessity of including individual as a random effect. Similarly, a separate model was fitted to test for the effect of season on monthly number of excursions. Significance of temporal autocorrelation was tested for in all models where monthly averages represented replicates for each fish.

Sea trout excursions from the Tvedestrand fjord to outer fjord and sea areas and to Østeråbekken were quantified and related to season. Excursions were defined as having a minimum length of 3 days. Additionally, the effects of length, body condition, and sex on time spent at sea were explored by linear modeling. The effect of length, body condition and sex on the probability of dispersing was assessed by a binomial generalized linear model (glm). We defined sea trout as dispersers if they left the study site within 2 months of tagging, followed by either not returning to the study site during tag life or spending >50% of their time outside the study area and river system. Dispersing sea trout were defined as receiving no protection from the reserve. Sea trout postsmolts have shown a low probability of migrating to sea if they did not exit the fjord within the first 41 days after leaving the river (del Villar‐Guerra et al., 2014); hence, sea trout that exited the fjord at a later stage were assumed to be expanding their home range beyond the fjord, rather than dispersing. To examine what proportion of the population is protected in the reserve, the proportion of time spent in the reserve given that the sea trout was in the fjord was multiplied with the proportion of time spent in the fjord by the tagged sea trout population as a whole. Here, dispersing sea trout were defined as spending no time in the fjord.

## 3 RESULTS

In total, 60 sea trout (mean body length: 34 cm, range 23–64 cm) were captured and tagged in the Tvedestrand fjord in 2013. Three individuals were excluded from the study due to postsurgical mortality (*n* = 1) and tag malfunction (*n* = 2). The remaining 57 fish generated 2,269,920 detections during the study, after removing false detections. The amount of time spent in the telemetry array by each fish ranged from 1 to 18 months (mean = 5.9, *SE* = 0.62).

**Figure 3:**
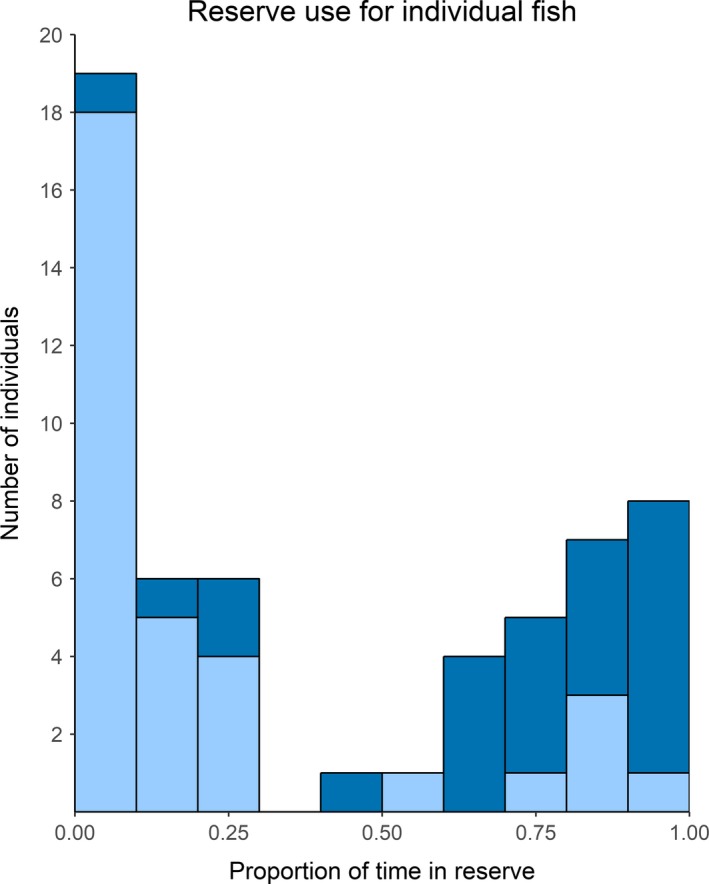
Distribution of the proportion of time spent in the reserve relative to time present in the fjord for all trout. Light blue and dark blue represent trout initially caught outside and inside the reserve, respectively

**Figure 4:**
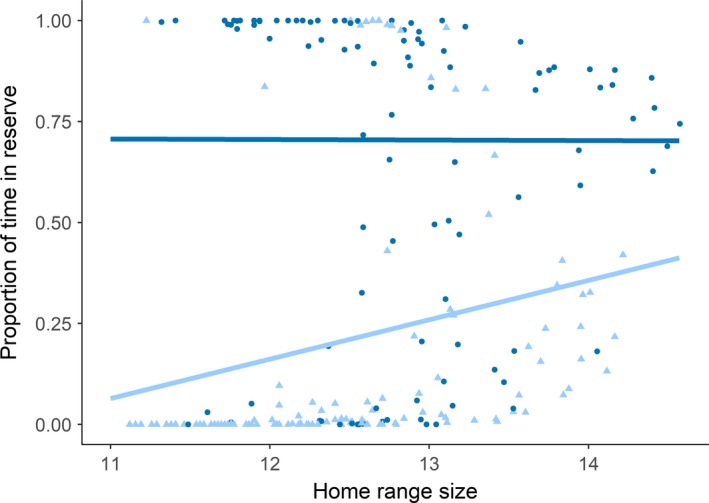
Proportion of time spent in the reserve plotted against 95% monthly home range size (log‐transformed). Light blue triangles represent observations from fish that were caught outside the reserve, while dark blue circles represent observations from fish that were caught inside the reserve. The light blue and dark blue lines show the predicted relationship between home range and proportion of time spent in the reserve for trout initially caught outside and inside the reserve, respectively

**Figure 5:**
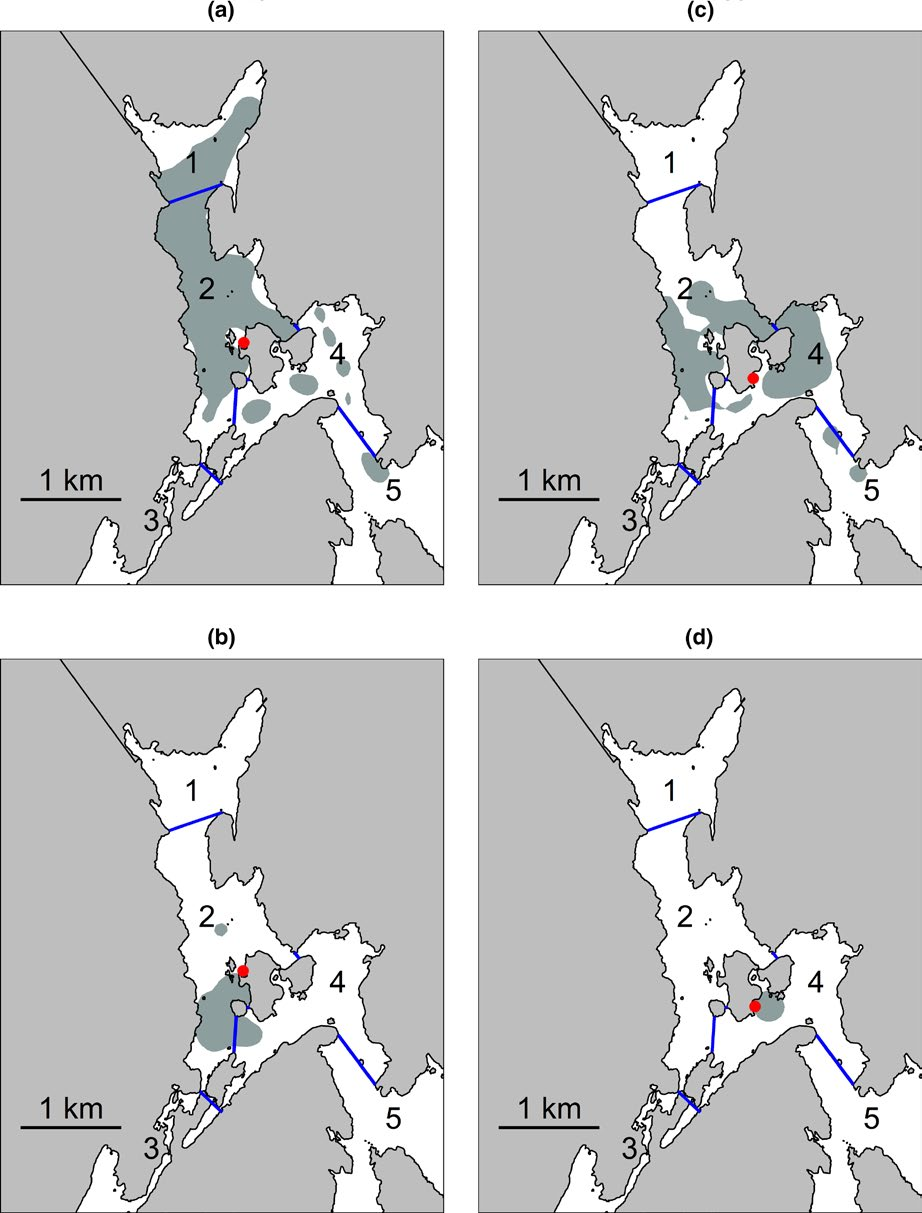
Examples of 95% home ranges of trout (a) with large home range caught inside the reserve, (b) with small home range caught inside the reserve, (c) with large home range caught outside the reserve and (d) with small home range caught outside the reserve. Blue lines delineate the zones, and red dots represent tagging locations. All home ranges are from May 2013 and selected among all tagged fish for illustrative purposes. The numbers on the map represent the different zones in the fjord

On average, sea trout spent 42.3% (±5.0% *SE*) of their time in the fjord inside the reserve (Table 1). Individuals utilized the reserve differently, with most trout spending either a large or a small proportion of their time in the reserve. Approximately half (53%) of sea trout spent less than 25% of their time in the reserve, whereas 33% spent more than 75% of their time in the reserve (Figure 3). Four individuals apparently did not visit the reserve during the study. The proportion of time spent in the reserve was not affected by fish length (*df* = 55, *p* = 0.240) or observation time (*df* = 55, *p* = 0.373). There was a marginally significant effect of season on time spent in the reserve (*df* = 334, *p* = 0.0574), where trout spent the least amount of time in the reserve during fall (34.4%) and the most in spring (46.0%). Furthermore, there was a significant interaction effect between home range size and capture location on the proportion of time spent in the reserve (*df* = 223, *p* = 0.0029). For trout captured within the reserve, home range size had a weak negative effect on proportion of time spent in the reserve (Figure 4). For trout captured outside the reserve, home range size had a stronger positive effect on proportion of time spent in the reserve (Figure 4). Including the identity of the trout as a random effect did not improve the model (ΔAIC = 1.88). Mean home range size was 0.430 km^2^, ranged from 0.0675 to 2.14 km^2^ (for examples, see Figure 5) and was not related to the number of PAVs calculated for a given month (*df* = 221, *p* = 0.106).

**Table 1:** Proportion of time (days) spent in the Tvedestrand fjord zones for all sea trout combined

Zone	Proportion ± *SE*
Zone 1	7.07 ± 2.14
Zone 2	42.3 ± 5.04
Zone 3	0.669 ± 0.547
Zone 4	47.9 ± 5.02
Zone 5	2.13 ± 0.985

Sea trout made an average of 0.38 (±0.052 *SE*) excursions from the reserve every day, and 92.8% of excursions were made to the central fjord area MPA (Zone 4). Movement out of the reserve occurred significantly more often during the day (60%) than at night (*p* < 0.001). Number of daily excursions was not affected by fish length (*df* = 51, *p* = 0.815), body condition (*df* = 50, *p* = 0.35), or sex (*df* = 46, *p* = 0.74). However, the monthly number of excursions was significantly affected by season (*df* = 322, *p* = 0.044). Summer and spring were the most different (*p* = 0.0765, *SE* = 1.89) with the fewest number of monthly excursions in summer (4.04) and the most in spring (8.59). Fish with larger home range sizes did more excursions from the reserve (*df* = 223, *p* < 0.001). Including the identity of the trout as a random effect did not improve the models.

The 15 individuals (26.3%) that utilized outer fjord and sea areas and returned spent on average 86.1 (±28.0 *SE*) total days at sea, and the average length of one excursion was 34.0 (±9.12 *SE*) days. Combined for all seagoing fish, there was little difference in time spent at sea in the different seasons (Table 2), and time spent at sea was not affected by fish length (*df* = 55, *p* = 0.115), body condition (*df* = 54, *p* = 0.28), or sex (*df* = 50, *p* = 0.21). Sea trout almost exclusively spent time in Østeråbekken during the spawning season in fall, with some stays extending into winter. Average total time spent in the river was 37.0 (±8.92 *SE*) days per fish, with the average duration of one excursion being 24.6 (±5.79 *SE*) days.

**Table 2:** Days spent at sea (*n* = 15) and in Østeråbekken river (*n* = 14) by season

Season	Days at sea	Days in Østeråbekken
Spring	313	5
Summer	311	0
Fall	328	403
Winter	339	109

A total of 35.1% of the sea trout were outside the study system at the end of tag life (*n* = 20), including the dispersed sea trout. Fish that dispersed to outer fjord and sea areas (*n* = 12) accounted for 21.1% of all tagged individuals. Fish length was close to having a significant positive effect on whether the trout dispersed from the fjord (*β*_Length_ = 0.56, *df* = 55, *p* = 0.0722). Body condition (*df* = 54, *p* = 0.21) and sex (*df* = 50, *p* = 0.67) did not affect dispersal. Time spent in the fjord by nondispersers was 96.6% (±1.4% *SE*), and the protection level afforded to all tagged sea trout by the current reserve was 32.3%.

## 4 DISCUSSION

This study evaluates factors determining the efficacy of a marine reserve for protecting anadromous brown trout. Overall, sea trout utilized the fjord extensively, spending only a quarter of their time in outer fjord and sea areas. While in the fjord, they spent on average 42% of their time inside the reserve, a proportion that corresponds to the size of the reserve relative to the study area. Sea trout caught within the reserve generally spent a larger proportion of their time within the reserve and for this group the effect of home range size on protection level was small, but slightly negative (Figure 4). In contrast, sea trout caught outside the reserve spent a smaller proportion of their time within the reserve and the effect of home range size was positive. Interestingly, this shows that home range size has a different effect on the amount of protection a sea trout receives from the reserve depending on capture location in the fjord.

Protection afforded by a reserve might be influenced by movement and home range size, with wide ranging and bold individuals experiencing less protection from a reserve (Parsons, Morrison, & Slater, 2010). There may be a heritable component to home range size and dispersal, implying that different genotypes may receive different levels of protection from a reserve (Harrison et al., 2015). Accordingly, based on a study of cod home ranges, it was theorized that having a larger home range could result in higher exposure to fishing outside the reserve and lead to fishery induced selection toward smaller home ranges (Villegas‐Ríos et al., 2016). In this study, we found that trout received a higher degree of protection with increasing home range size if initially captured outside the reserve (Figure 4). The different response to increasing home range size for individuals tagged within and outside the reserve indicates that if selection pressure toward smaller home ranges was to exist within the reserve, it can be opposed by the individuals outside the reserve. However, the selective landscape must be seen in concert with the selection pressure inflicted by angling in itself. Angling has been shown to select against boldness in carp (*Cyprinus carpio*) (Klefoth et al., 2017), and Alós et al. (2016) show that pearly razorfish (*Xyrichthys novacula*) individuals characterized by a high exploration intensity and a large home range radius are quickly removed from the population when exposed to an intense angling fishery. In total, abundance was reduced by 60% within a few days. In the present study, we do not present rates of fishing induced mortality and can thus only comment on the potential for protection within reserves. Also, potential selection on home range sizes within and outside the reserve may be limited if the tagged trout originate from different populations. We tagged sea trout within a limited area and assumed that most individuals belonged to the same gene pool.

Length, body condition, and sex of sea trout were not related to movement at sea, but size was close to having a significant positive effect on dispersal. The latter is in line with findings by Flaten et al. (2016) and Bordeleau et al. (2018), showing that female sea trout migrating to the outer fjord areas were larger than females migrating to inner fjord areas in Norwegian fjords. In contrast to our findings, an earlier study found that low body condition correlated with increased migration distance in sea trout, potentially for the purpose of maximizing feeding opportunities (Eldøy et al., 2015). Haraldstad et al. (2018) also found that poor condition correlated with an extended marine stay and skipped spawning migrations in sea trout in Skagerrak. Furthermore, home range size has been shown not to correlate with size for trout (Závorka, Aldvén, Näslund, Höjesjö, & Johnsson, 2015), and it has also been shown that migratory decisions in the fjord are not affected by size (del Villar‐Guerra et al., 2014). Overall, our results imply that the reserve does not inflict a size‐selective protection regime on the sea trout population within the fjord. In our study, the potential selectivity of the sampling location must be taken into account, as sampling was only conducted around the islands in the center part of the fjord (Figure 2) and not in the river or outer fjord and sea areas. Individuals that disperse from the fjord within a short time frame are less likely to have been sampled, and the length distribution and body condition of these fish is unknown. In general, individuals and behavioral types that mainly utilize the inner parts of the fjord or the outer fjord and sea areas are less likely to have been sampled.

Excursions from the reserve were mainly to Zone 4, which comprises the central fjord MPA. Movement between these zones is likely to represent random movements within a home range. However, the relatively few excursions to Zone 1, combined with the low proportion of time spent there (Table 1), may indicate that sea trout find the area outside the river inlet less favorable than the central part of the fjord. This may be related to higher availability of food further out in the fjord which has previously been suggested as a migratory decision characteristic (del Villar‐Guerra et al., 2014) and an explanation for trout to spend less time in inner fjord areas (Morris & Green, 2012). Previously, low biodiversity has been observed at sampling stations in Zone 2, close to the border between Zone 1 and 2, indicating a reduced selection of prey for sea trout in this habitat (Kroglund, Dahl, & Oug, 1998). More likely, the low proportion of time spent in the inner part of the fjord is due to no individuals being tagged in this region. There were significantly more excursions from the reserve during day than night, implying greater horizontal movement during day. Salmonids have shown great differences in movement rates contrasting day and night (Alanärä, Burns, & Metcalfe, 2001; Candy & Quinn, 1999; Eldøy et al., 2017; Goetz, Baker, Buehrens, & Quinn, 2013), and it has been shown for steelhead trout (*Oncorhynchus mykiss*) that horizontal movement rates increase twofold during daylight compared to night in the marine habitat (Ruggerone, Quinn, Mcgregor, & Wilkinson, 1990). This may lead to a higher exposure to fishing during the day.

Sea trout resided in Østeråbekken stream almost exclusively during spawning season in fall, including some extended stays into the winter season. Also, sea trout spent significantly less time in the reserve during fall. This confirms the theories about spawning behavior previously documented for sea trout (Klemetsen et al., 2003; Knutsen, Knutsen, Olsen, & Jonsson, 2004; Olsen, Knutsen, Simonsen, Jonsson, & Knutsen, 2006).

Following the predictable spawning migration of sea trout, it can be expected that individuals receive protection from the reserve in the fjord while migrating to and from river spawning areas. A study on Arctic charr (*Salvelinus alpinus*) showed that an MPA located in a fjord, also encompassing the nearest spawning river, on average protected the tagged population one‐third of the time (Morris & Green, 2012). In the present study, there were seasonal differences in reserve use, with sea trout spending a larger proportion of time in the reserve and performing most excursions from the reserve during spring, the latter indicating more horizontal movement in this period. Furthermore, protection extends to straying trout that arrive in the spawning river. In a study of how stocked sea trout uses nearby rivers, Degerman et al. (2012) suggest straying rates were twice as frequent for individuals stocked in small rivers as a consequence of less available habitat. Overall straying rates (including nonspawners) of up to 57% were observed, and temporary use of non‐natal rivers occurred more often in large rivers (Degerman et al., 2012). This indicates that situating reserves in fjords with large spawning rivers may increase the number of individuals that receive protection from the reserve, and thus also protect individuals from nearby river and fjord systems during migrations. Further studies may reveal more detailed habitat preferences in sea trout, but previous studies indicate that individual fish exhibit highly variable movement patterns in marine areas (Middlemas, Stewart, Mackay, & Armstrong, 2009). However, sea trout have shown slower rates of movement away from spawning rivers than salmon (Finstad, Økland, Thorstad, Bjørn, & McKinley, 2005; Thorstad et al., 2007), thus spending more time in the fjord may improve protection by reserves.

Given their broad distribution and desirability in fisheries, there are a range of areas where implementation of reserves may be useful in maintaining sea trout populations. For example, populations are threatened by overfishing such as in the Gulf of Bothnia and the Gulf of Finland in the Baltic Sea (HELCOM, 2011). In these regions, sea trout are bycatch in other fisheries, such as whitefish and pikeperch, and fishing mortality may reach 80%. With high mortality rates occurring in fisheries, protection of fjord based populations or spawning areas may be crucial to sustaining sea trout populations.

In conclusion, this study revealed that even a relatively small no‐take marine reserve has potential to protect the full home range of sea trout displaying small to intermediate home range size while residing in the marine habitat. Furthermore, sea trout initially tagged in the reserve received more protection than individuals tagged outside the reserve, while individuals tagged outside the reserve received more protection with increasing home range size. This attribute of the no‐take/partially protected zone mosaic studied herein can potentially oppose the combined effects of “protection‐induced selection” toward smaller home ranges within reserves—and angling‐induced selection toward less bold behavior and smaller home ranges outside reserves. From a selection perspective, MPA and MPA network design can affect the selective landscape through which sea trout are moving during the marine phase. This perspective has important evolutionary implications for marine reserve and MPA network design. Although “Darwinian MPA design” requires good knowledge regarding key features of target species' movement ecology and life histories, it is worthwhile to develop design criteria that will improve the protective qualities of spatial management measures and ensure long‐term benefits to protected populations.

## AUTHOR CONTRIBUTIONS

All authors were involved in conceiving and developing ideas and designing methodology; EMO and EM collected the data; SHT analyzed the data and led the writing of the manuscript. All authors contributed critically to the drafts and gave final approval for publication.

## DATA ACCESSIBILITY

Data is available from the Dryad Digital Repository: http://doi.org/10.5061/dryad.fq3d127
