# Supplementary material for: Potential of a no‐take marine reserve to protect home ranges of anadromous brown trout (Salmo trutta)
Source: Ecol Evol. 2018 Dec 18;9(1):417–26. doi: 10.1002/ece3.4760 (PMC6342106; doi:10.1002/ece3.4760)
Supplement: Supplementary file 1 [file ECE3-9-417-s001.docx]

**Supplementary material**

To identify if there was a higher detection probability in the reserve than other zones due to its higher density of receivers, time intervals between position averages (PAVs) was calculated for all zones separately and compared (Fig. S1). Minimum time interval between one PAV and the next is 30 minutes. Time intervals longer than 12 hours (720 minutes) were excluded, as they were more likely to represent a fish that left the zone than a fish undetected by the receivers.


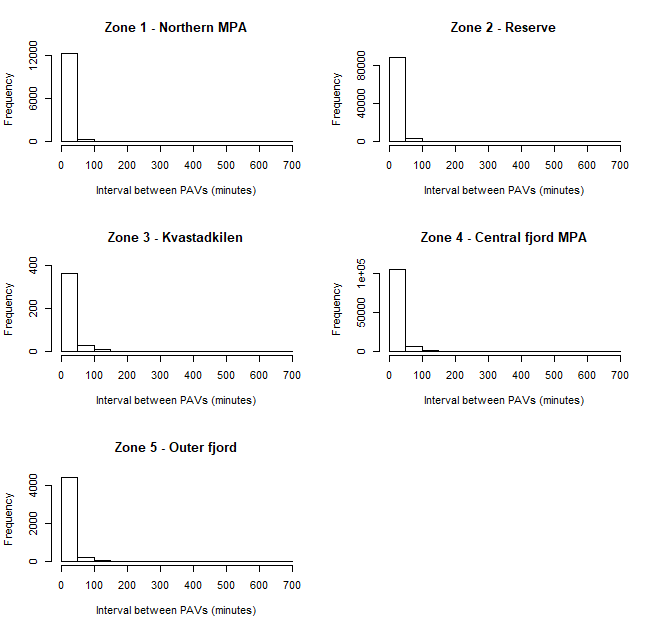


**Figure S1:** Distribution of time intervals (minutes) between each calculated position average (PAV) in the 5 zones of the fjord.
